# Interdisciplinary Approach to Biological and Health Implications in Selected Professional Competences

**DOI:** 10.3390/brainsci12020236

**Published:** 2022-02-08

**Authors:** Dorota Kostrzewa-Nowak, Robert Nowak, Joanna Kubaszewska, Waldemar Gos

**Affiliations:** 1 Institute of Physical Culture Sciences, University of Szczecin, 17C Narutowicza St., 70-240 Szczecin, Poland; robert.nowak@usz.edu.pl (R.N.); jo.kubaszewska@gmail.com (J.K.); 2 Institute of Economy and Finance, University of Szczecin, 64 Mickiewicza St., 71-101 Szczecin, Poland; waldemar.gos@usz.edu.pl

**Keywords:** behavioral genetics, neuroaccounting, personality

## Abstract

Everyday life’s hygiene and professional realities, especially in economically developed countries, indicate the need to modify the standards of pro-health programs as well as modern hygiene and work ergonomics programs. These observations are based on the problem of premature death caused by civilization diseases. The biological mechanisms associated with financial risk susceptibility are well described, but there is little data explaining the biological basis of neuroaccounting. Therefore, the aim of the study was to present relationships between personality traits, cognitive competences and biological factors shaping behavioral conditions in a multidisciplinary aspect. This critical review paper is an attempt to compile biological and psychological factors influencing the development of professional competences, especially decent in the area of accounting and finance. We analyzed existing literature from wide range of scientific disciplines (including economics, psychology, behavioral genetics) to create background to pursuit multidisciplinary research models in the field of neuroaccounting. This would help in pointing the best genetically based behavioral profile of future successful financial and accounting specialists.

## 1. Introduction

The financial world is the basis for the contemporary economy, and business and finance play a key role in societal development. Thus, determining those personality traits that are the most useful in various areas of financial activities would seem to be of key importance. With respect to economics, accounting, including financial or management accounting, plays a fundamental role in shaping an enterprise’s financial and management politics, no matter how large it is. Accounting is the basis of the contemporary business world, meaning that an accountant must possess extensive knowledge of law, economics, and finance. Moreover, the expertise that any specialist in accounting must possess includes not only the ability to interpret and solve complicated legal problems but also to work under the pressures of limited time and significant stress.

All the aforementioned factors make the accounting occupation interesting with respect to behavioral sciences, a field of research that encompasses interdisciplinary studies spanning biology, psychology, medicine and social sciences including economics. As this field may examine the influence of psychological, societal, and emotional factors on financial decisions and their consequences, it has assumed crucial importance in recent years. One of the most frequent definitions of neuroeconomics claims that it is the science studying the influence of brain and the nervous system on economic behaviors and is strongly related with psychological sciences. Behavioral research aims at defining the actual process of deciding, causing an increase in in financial reporting effectiveness as an instrument of data communication [1]. For some time, neuroaccounting [2,3,4,5,6] has been gaining in popularity within the specialist literature, demonstrating that the academics have noticed the essential role that neurobiology can play in shaping expertise essential to the financial sector. Despite the fact that the biological mechanism associated with susceptibility to financial risk has been very well described [7,8,9,10,11,12,13,14,15], little or no data explain the biological basis for neuroaccounting. As accountants form a very large part of the active workers in every population and their job market is extensive, it is worthwhile to define the inheritable traits that can play a significant role in the success of workers connected with accounting at different stages of their careers.

The cognitive neuroscience method and the neuroaccounting theory are used to explain the behavior of enterprises in terms of decision-making within corporate social responsibility [5,16]. So far, researchers have provided explanatory data identifying functional networks that constitute distinct and vast areas of the nervous system that work together to coordinate cognition and behavior [17,18]. Thus, the reward network is related to responses to pleasant stimuli and thus to learning and motivation processes; the control network is related to focusing attention, processing information and identifying behavior consistent with the goal and the default mode network is associated with resting state processes such as self-discovery, reflection, and automatic responses derived from habits or experiences [18,19,20,21]. When analyzing the meaning of accounting and the range of competences that are necessary in the accounting profession, it is assumed that accounting is action-oriented, the purpose of which is to influence the action (behavior) directly through the information content of the communicated message and indirectly through the behavior of accountants [22]. Behavioral accounting aims to improve the understanding of decisions made by participants of the accounting system in the production of accounting and non-accounting information that is influenced by reports and accounting statements [3]. Research in behavioral finance and behavioral accounting fields help to understand the relationship between decision-making processes and psychological aspects in finance and accounting. Although these modes use cognitive neuroscience, they are often devoid of biological background at the cell signaling level combined with behavioral genetics.

From this point of view, the neuroeconomic sciences, including neuroaccounting should also take both the genetic and environmental factors as important predictors to create a novel research model into account.

In 2018, the Polish Accountant Society prepared a report entitled “Portrait of an Accountant,” which discussed the main characteristics of this workgroup and answered the basic question: Who are accountants? Poland’s economic workforce encompasses around 200 thousand people working in the financial or accounting field. Around 70.3% of surveyed accountants were reported as finding their occupation “prestigious”. Like educational, social, and financial factors, prestige, which is synonymous with the words esteem, admiration, and appreciation, defines a clear hierarchy of more and less important people. Unlike measurable parameters, prestige expresses self-awareness, is associated with increased self-esteem and better self-assessment, and can induce people to have predetermined attitudes. Among entrepreneurs and contractors, 44.9% perceive accountants as trusted advisors, and 44.7% describe them as financial guardians and 42% as high-class specialists, especially among accountants above 50 years of age with extensive work experience. How then do accountants view themselves? Which traits should a person possess to enter the economic workforce as an accountant? 74.3% of respondents listed meticulousness as the most important quality, followed by personal integrity. Next in order of popularity were diligence, desire to improve, stress resistance, creativity, inquisitiveness, assertiveness, decisiveness, and a strong work ethic. Accountants often work under time pressure and have to process a large amount of data, requirements that call for knowledge, analytical skill, and abstract thinking ability. In addition, survey participants also mentioned the following additional qualities: “nerves of steel, the gift of the gab, awareness of their own lack of knowledge and that of those around them, availability, multitasking, divisibility of attention, ability to see the big picture, patience, ability to explain complicated matters, flexibility, good memory, imagination, honesty, systematics, ability to make something out of nothing, discretion, communicativeness, self-organization and self-discipline, prescience, understanding (to lack of knowledge), trustworthiness, mind-reading abilities, ability to listen, attractiveness, propriety, resistance to pressure, intelligence, cleverness, passion for one’s art, diplomacy, humanism, specific knowledge, perseverance, ambition, composure and ability to distance oneself, cheerfulness, loyalty, business thinking, and availability” [23]. The qualities required by accountants described in literature data are divided into three areas:

(i) Knowledge: business processes, financial accounting and law, use of IT tools, companies environment;
(ii) Skills: analytical thinking, interpretation of financial and non-financial data, communication skills, ability to work in a team, strategic thinking;
(iii) Personality trait: work under pressure, adaptation to changes, ethical behavior, decision making [24,25,26]. The lack of these qualities puts one’s ability to succeed as an accountant open to question.

## 2. The Biological Process of Decision-Making

Behavioral research methods focus on units; groups and even whole organizations are often used to understand human attitudes. Procedures such as laboratory examinations (in which participants complete a task under supervision in a laboratory setting); surveys exploring participants’ characteristics (e.g., life views, attitudes, cognitive processes or motivations); an experimental economy that encourages a transaction between a buyer and seller in an artificial market setting; field experiments, where participants work under normal conditions, are commonly used. In particular, laboratory examination is very useful in understanding individual differences [27]. A huge advantage of behavioral research methods employed in laboratory examination is that external factors can be eliminated, making determining a correlation between causes and results possible. This type of research aims to analyze the influence of external factors, but, to better understand the whole decision-making process, one needs to analyze the thought process.

The ’90s and the start of the 21st century marked a period of intensive progress in brain imaging and hence a greatly improved understanding of how it functions. Definite breakthroughs occurred during this time, not only in medical science but also in many areas that had previously been thought to have little or nothing to do with biology. One of these was neuroeconomics, which combined the findings of psychology, neuroscience, and economics, and so permitted broad, multi-aspect research into the complex occurrences happening during or just before decision-making. Thus, through analysis, understanding, and the ability to interpret thought processes, situations connected with money, investment, management, and assuming financial risk could be studied [2,3,4,5,6,7,8,9,10,11,12,13,14,15,28].

The most effective methods for scanning brain activity are functional magnetic resonance imaging (fMRI), which can locate the areas of the brain involved when a subject is performing certain tasks; diffusion-weighted magnetic resonance imaging (dMRI), which images sections of the brain; electroencephalography (EEF), which records electrical activity of the brain; positron-emission tomography (PET), which registers gamma rays emitted indirectly by a positron-emitting radioligand introduced into a subject’s body; tomography; and transcranial magnetic stimulation [4,29]. These tools explain and illustrate various reactions of the brain on the neuronal level and are effective in examining automatic and unconscious mental processes such as those determining behavior related to economics. In situations where subjects experience enjoyment from undertaking and settling decisions following rational analysis, surveys and interviews are not very efficient research methods. However, thanks to the methods listed above that enable scanning of the brain, researchers have been able to create an “economic brain map” [28].

To fully understand thought processes, one must first understand the brain’s structure. The two cerebral hemispheres, while similar in appearance, differ in function and structure. The right hemisphere is responsible for creativity and intuition and plays a decisive role in linguistic tasks; analysis of the prosodic aspect of language; understanding metaphors; and humor. On the other hand, the left hemisphere is the site of mathematical and visuospatial ability [30,31,32,33]. Moreover, the human brain can be divided into two systems with respect to type of work–an automatic system located under the cerebral cortex that creates emotions and reflexes (basal nucleus and limbic system) and an analytical system that is involved with rational thinking, planning, and analyzing and that connects with the prefrontal and parietal cortex. The analytical system is relatively “new” because it originates from the formation of the neocortex, whose layers grew to overlap the “old” areas of the brain during the evolutionary process. The reflex-based brain is quick, automated, and entirely unconscious, as when we draw back our foot after stepping on a pin. Reflex movement occurs before our consciousness can notice and acknowledge the stimulus. The part of the brain responsible for reflex movements avoids overworking itself, and so it avoids negative stimuli and reacts to pleasant ones. In a situation with which the reflex-based brain cannot cope, the cognitive brain steps in; it is more rational and is able to interpret emotions [34]. In decision-making, the human factor motivating us to prioritize our survival is usually the most important one. Therefore, most of an investor’s decisions are not consistent in a logical sense but are understandable from an emotional perspective.

Financial decisions involve forecasts for data in the future, statistics, and personal anticipations about risk and its consequences, and this type of situation can cause an emotional whirlwind clouding the ability to think logically because of its strong emotional impact, possibly a storm of conflicting, potent emotions like hope, happiness, and confidence but also fear, panic, greed, amusement, or even grief. According to Zweig [34], neuroeconomic research illustrates the principle that optimum results are achieved when emotions are controlled, but not eliminated completely since lack of emotions can be destructive.

## 3. Behavior and Personality Type

Human behavior results from the total of one’s coordinated personality traits formed through adolescence and biological, mental, societal, and spiritual development. It regulates and integrates activities, gives direction to social behavior by imposing certain attitudes, and teaches one how to direct oneself. Personality can be described according to five primary, independent determinants spanning a large spectrum of traits: extroversion, agreeableness, conscientiousness, neuroticism, and openness to experience. According to Hippocrates, an ancient Greek physician and philosopher and the first known researcher in human temperament and behavior, there were four temperaments.

The first type, the sanguine personality, is energetic, well-balanced, and strong. Capable, healthy, and always alert, when deprived of stimuli it calms or even hibernates. Pavlov considered this type as the optimal, as it is difficult to cause this personality type distress or mental breakdown. The second one, the phlegmatic personality, is strong and balanced but, unlike the sanguine one, is very slow. The phlegmatic nervous system adjusts well to conditions in order to survival. Strength and balanced braking and excitation processes increase resistance to stress in this type, resulting in difficulty with inducing anxiety. A phlegmatic’s emotions are always constant, but this type has difficulty in adjusting to rapid changes. The third type, the choleric, is strong but unbalanced. Excitation processes start fast, while inhibitory processes are reluctant. It causes excitation processes to overshadow inhibitory ones, resulting in a prevailing sense of anxiety. Choleric types find it difficult to abstain from doing something. In difficult situations which require strong braking mechanisms, cholerics experience high anxiety, often resulting in exhaustion and sometimes leading to insomnia and depression. In some situations, their anxiety results in aggression. The fourth type is melancholic, described as weak. A positive, conditioned response builds up slowly under external conditions and is susceptible to weakening and atrophy. There is very little resistance to the braking process, and so when a melancholic has to abort a task, disorganization results. The melancholic personality is ill-prepared for life’s challenges, is non-combative and susceptible to breakdowns, suffers from anxiety and mental illness, and avoids making bold decisions and leaving a comfort zone [35].

C.G. Jung proposed two primary attitude types: extraversion and introversion. Extroverts focus on the outside world, connect with people, and tend to be loud and dynamic. They adjust to changes more easily, are expressive, witty, and talkative, and have a distinct style of nonverbal communication. Introverts, on the other hand, tend to focus on themselves, are inhibited in voicing their opinions and tend to be insensitive to the outside world. Their lives are less noisy and take place in the context of close relatives and/or friends. They tend to achieve greater academic success and are less prone to addiction and crime. They are pleasant-natured and less aggressive but withholding emotions can make them susceptible to anxiety.

The personality types described above act as a stencil, outlining the ideal state of the beforementioned personalities. Human personality is often, in fact, a mix of some or all of the aforementioned qualities, with one trait predominating, and it can serve as a basis for personality analysis, performed through the structure of a temperament questionnaire (STQ) or personality inventory. Because of the level of complexity in human personality, it is difficult to predict behavior and aptitudes based on the personality traits of a worker, which increase the difficulty associated with human resource management. In addition, not every personality type suits every type of work. For instance, introverts fare better as accounting and finance specialists, administrative specialists, and cost estimators, whereas extroverts excel as marketing and sale associates, lawyers, engineers, and sales representatives. Melancholics are great accountants and economists, sanguines make excellent sales representatives and marketing specialists, phlegmatics make perfect accountants, and choleric are superb engineers [36].

Another popular methodological concept is Cloninger’s Psychobiological Model of Temperament. The basis of this theory [37,38,39,40] is the assumption that neurotransmitters can influence certain aspects of human personality [41]. According to this theory, human personality encompasses both genetic and environmental factors. In Cloninger’s theory, novelty-seeking, harm avoidance, and reward dependence are temperamental dimensions that have direct biological implications. Novelty-seeking temperament has a direct link to the dopaminergic system, harm avoidance to the serotonergic system, and reward dependence to the noradrenergic system [37,38,39,40].

Variety of personality theories based on different factors raise question about the criteria that should be used to select the most research optimal model. The medical criteria of mental and personality disorders are classified according to World Health Organization in International Statistical Classification of Diseases and Health Problems (ICD) as well as created by the American Psychiatric Association DSM classification. Barrick et al. [42,43] used five-factors (extraversion, emotional stability, agreeableness, conscientiousness, openness to experiences) models of personality and evidenced relations between personality and job performances. On the other hand, this meta-analysis inspired Cewińska et al. [44] to perform a study of personality profile among finances and accounting students using Holland theory [45,46,47]. None of these theories had taken the neurobiological implication being key important in neuroaccounting study into account. One of the psychobiological models that can be used in neuroaccounting is the Cloninger’s personality model. The base of this theory [37,38,39,40] is the assumption of the modulating effect of the neurotransmitter system on the expression of specific features of human personality [41]. The Cloninger’s theory describes both genetic and environmental factors as a key important to determining personality. According to this theory, there are four dimensions of temperament: novelty seeking (tendency to actively respond to new incentives), harm avoidance (tendency to inhibit actions in response to negative incentives), reward dependence (tendency to maintain behavior in response to positive incentives) and persistence (ability to independently support a given type of activity). Three of them have a strong neurobiological implications: novelty seeking (PN), harm avoidance (US) and reward dependence (ZN). PN temperament is associated with the dopaminergic system; US-with serotonergic system, ZN-with adrenergic system [37,38,39,40,41,42,43,44,45,46,47,48,49,50].

On the anatomical and physiological level the main dopaminergic neuronal pathways leave the midbrain and run to the forebrain. Dopaminergic neurons located in the abdominal part of the cap give projection into limbic structures creating a motivational system [48,49] and because of that, on the psychological level, it is related to behavioral approach system in the Cloninger’s theory [50,51]. Dopamine is the main neurotransmitter in this system. This catecholamine is a ligand for dopamine receptors (DRD 1-5) and is transported by dopamine transporters (DAT). An attempt to interrogate links between dopamine and human phenotypes resulted in numerous studies reporting statistical associations between single nucleotide polymorphisms (SNPs) or other polymorphic variants in dopamine genes and a phenotype of interest, such as performance on a cognitive test [52], a neuropsychiatric diagnosis [53], or a neuroimaging measure [54]. The dopamine signaling diagram is presented in [55]. On the other hand, the synthesis of catecholamines is observed not only in nervous system therefore the environmental factors like perceptual-cognitive takas may influence on both paracrine and endocrine secretions of these ligands. 

**Figure 1:**
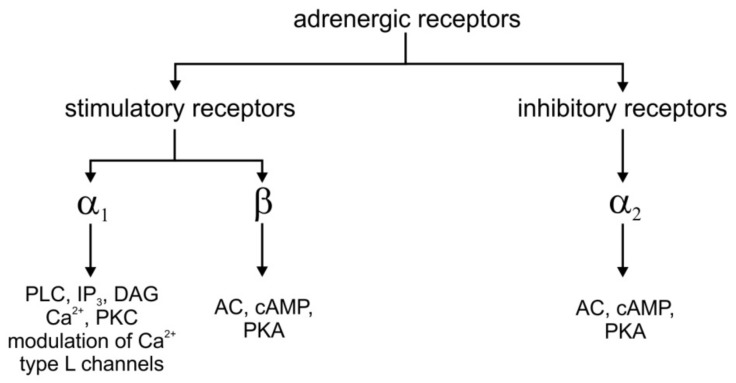
Classification of adrenergic receptors [56,57,58]. cAMP—3’,5’-cyclic adenosine monophosphate, DAG—diacylglycerol, IP3–inositol 1,4,5-trisphosphate, PKA—protein kinase A, PKC—protein kinase C, PLC—phospholipase C.

Adrenergic neurons are found in the pons and medulla oblongata and their largest concentration is the locus coeruleus [48]. The brain adrenergic system is involved in a variety of behaviors including: agitation, attention, wakefulness, anxiety, reactivity to stress stimuli, learning and memory processes [49]. The receptors for noradrenaline and adrenaline are heterogeneous groups classified according to pharmacological criteria and indicated different molecular signaling mechanisms (Figure 1) [56,57,58]. It must be emphasized that the expression of adrenergic receptors is common in different tissues e.g., muscles (both heart and smooth ones) [59], fat tissue [60], or leucocytes [61]. On the behavioral level, the participation of this adrenergic system is known as a fight/flight system responsible for unconditional aversive reactions [50].

It is well known that serotonin is essential for the maintenance of synaptic plasticity, motivational and reinforcement processes [62,63]. The central serotonergic system regulates mood and has been implicated in several neuropsychiatric conditions and behavior [64]. The pharmacological studies evidenced that serotonin receptors (5-HT) are proteins involved in various neurological and biological processes, such as aggression, anxiety, appetite, cognition, learning, memory, mood, sleep, and thermoregulation [65]. The classification and molecular mechanism of biological activity of serotonin receptors are presented in [66].

Brain-derived neurotrophic factor (BDNF), similarly to serotonin, plays an important role in neurogenesis and brain neuroplasticity. It evidenced that BDNF is a strong modulator of synaptic transmission, and its activity is also related to learning and memory processes [67]. The molecular mechanism of BDNF activity is related with high affinity with a receptor belonging to the family of kinases associated with tropomyosin (TrkB). Intracellular signaling stimulated by BDNF/TrkB is crucial for neuronal survival, morphogenesis, and plasticity [68]. BDNF seems to be an important factor involved in increasing of cognitive skills because of its biological role. The reflection of BDNF neuronal secretion is the measurement of the concentration of this protein in the serum or plasma of peripheral blood [69].

Neuropeptide Y (NPY) is a highly conserved endogenous peptide in the central and peripheral nervous systems of all mammals, which has been implicated in both pro- and antinociceptive effects. NPY is expressed in the superficial laminae of the dorsal horn of the spinal cord, where it appears to mediate its antinociceptive actions via the Y1 and Y2 receptors [70]. NPY biological effects are often affected in mood disorders, including the modulation of neuronal activity (glutamatergic, adrenergic, serotonergic, GABAergic and dopaminergic), influence circadian rhythms, memory processing, feeding, alcohol consumption, and the hypothalamic-pituitary–adrenal axis [71]. From this point of view, this molecule may be involved in cognitive fatigue and stress-related molecular pattern. It is worth knowing that the biological role of NPY is well described using animal models [72]. 

The abovementioned neurophysiological mechanisms are often discussed in medical and biological science. There are no psychosocial research in the aspect of neurobiological mechanisms of neruoaccounting. Moreover, to our best knowledge, there are no study explaining relations between neurophysiological and molecular (on both genetic and neurobiochemical levels) aspects of neuroaccounting. Even though there are numerous data in the field of behavioral economy describing psychological aspects of making financial decision and some genetics correlations were also observed, no attempts have been made to determine biological changes associated with the release of signaling factors affecting the functioning of the nervous system into peripheral circulation as well as metabolic changes arising as a result of psycho- and sensorimotor stimuli that mimic the cognitive competences required form specialists in finance and accounting. It is worth emphasizing that research conducted in the field of perception and cognitive assessment is characterized by more effective collection and coding of relevant information, more effective use of available information by making optimal decisions, as well as faster information processing and adaptation in learning [73,74,75,76,77,78]. The cognitive skills are often evaluated by assessment of brain activity through endogenous event-related potentials related to the cognitive processes and is undertaken with the help of cognitive tests. 

Relationships between paracrine and endocrine secretion of signaling molecules, e.g., catecholamines (including dopamine, adrenaline), serotonin, cortisol, BDNF, NPY and the influence of perceptual-cognitive tasks describes the neurobiological mechanisms that develop under the influence of specific stimuli. In this area the knowledge of neurological mechanisms at the physiological and molecular level gives a broader understanding of the issues of neuroscience. It also shows the importance of life sciences in understanding research on cognitive plasticity and professional adaptation in a variety of professions, including those related to finance and accounting. It seems that the perception of neurobiological dependencies by the economic sciences community may be deepened by cellular mechanisms enabling a deeper understanding of cognitive processes related to professional work. The interdisciplinary research in the field of behavioral biology (on genetic and cytophysiological level) allows to link the influence of psychological, emotional, cognitive and physiological factors on the resultant functional response, which translates into general health of person.

## 4. Behavioral Genetics and Trait Inheritance

In the decision-making process, the initial reaction is to appeal to emotion and intuition and to individual thinking instead of rational analysis of data, which, sadly, does not always come have a hand in rational decision-making. In addition, the financial world can have serious consequences to the computability and predictability of economic realities. It forces us to seek new strategies and develop fresh views of a problem [79]. The following questions thus arise: Is emotional behavior only an element of the main factor, which is environmental, and the challenges it imposes? Or does human sensitivity have tangible genetic foundations? Or perhaps both viewpoints are true?

Feelings and emotional processes are an inseparable part of thinking and decision-making. They are regulated by hormones and a series of biochemical processes of the human body. Extensive research based on psychological tests and molecular techniques has produced significant data about the genetic foundations of human behavior and shaped a new scientific field–behavioral genetics, a scientific field which embraces concepts stemming from social sciences and genetics and which focuses on within-population differences in personality, intelligence, behavior, thinking, and associated disorders. People vary in certain, significant ways with respect to common individual differences. Each of the traits, for example, openness to new experiences, or sensibility, is measurable. Test questionnaire results can, for instance, indicate whether a test subject is strongly or low-emotional. Behavioral geneticists aim to ask the questions: How significant are the influences of genetic and environmental factors on the general behavioral variability in a specific population? Is there any connection between genes and human behavior? Are changes in genetic coding correlated with behavioral changes?

One of the most common research methods is a twin study, which focuses on researching behavior in twin pairs, who can have grown up together in one family or apart in separate families due to adoption or divorce. A second popular method is a family study, which aims to compare behavior between parents and their children. A third method compares the behavior of adoptive children to that of adoptive parents. Molecular genetics is the newest and most undeveloped research method. It helps to identify genes and examines their level of polymorphism. It also imposes the questions: Where are those genes located, how many of them are there, and what are their function? After long-standing research, we can conclude that the variability observed in most features is polygenic, and the more elaborate a feature is, the more genes have had a role in influencing its shape. Feature variability can be divided into two groups–additive, related to parental influence, or nonadditive (nonhereditary), associated with gene interaction within an organism (epistasis, domination). Thus, gene expression products shaping some features interact with other gene expression products, resulting in a particular trait developing differently than it should or being silenced altogether. All the aforementioned tests aim to define heritability, which is a statistical method used to measure gene influence on trait development.

The source of behavioral variability lies in the environment and in social interactions. According to a test conducted on adult pairs of twins living together, the longer they shared a living space, the more similar they became. The following questions then arise: If we place ordinary children in a class with people presenting very high mathematical skills, would they be able to also gain this proficiency due to gaining similarity with their gifted classmates? Would social interaction reinforce those mathematical proficiencies? Environmental tests can distinguish between two types of genotype-environment correlation (GE correlations): the active type, in which a person possessing the genotype searches for the most suitable environment in which to express inherited traits, thus influencing career path decisions, and the reactive type, which represents a reaction to behavioral traits determined by inheritance, thereby reinforcing the trait. This assumptions means perfect environments can be created to develop desired traits. For instance, the development of a child that displays a proficiency in arithmetical calculations could be helped through stimulation and partaking of a beneficial environment or even by the child’s being given beneficial tasks (e.g., counting pocket change during grocery shopping). The second type correlation is known as the genotype and environment interaction (GxE) type [80,81,82,83,84].

## 5. Stress Resistance

Stress is a factor of human existence, both in private and in work life. The National Institute for Occupational Safety and Health defines stress as a situation where external demands are perceived by the individual as incompatible with their abilities or needs. Thus, work-related stress represents discrepancies between environmental demands and employee abilities, and time pressure can increase feelings of fear and stress [85,86,87]. The impact of stressful situation on immunity is widely discussed in the literature [88,89,90,91,92,93]. Psychological stress induces an immune signaling both in healthy and sick people [94]. Physical, cellular and psychological stress initiate the release of endogenous factors known as danger- or damage-associated molecular patterns (DAMPs) to promote sterile inflammation, the activation of inflammation processes in the absence of exogenous factors such as pathogens [90,91,95,96,97,98]. Better understanding of immune signaling pathways activation among healthy working women and men and correlation them with each individual’s psychological profile and cognitive skills might be of key importance in creation modern health-related program helping to monitor stress in the work environment. However, it needs broad research combining different scientific disciplines.

An accounting job demands a very specific work ethic. Time pressure is sinusoidal, because reporting deadlines and invoice settlements tend to happen in cycles, meaning that, when the billing period starts, work piles up and, when a billing cycle ends, the amount of work and stress decrease substantially. We can classify stress as positive–motivating–and negative–debilitating, demotivating, having serious social consequences and damaging to one’s health. Reaction to stress is individual and depends on one’s intellectual, emotional, and physical abilities [99]. According to Pietrzak& Wnuk-Pelal, research among the personality traits desirable in the accounting profession the highest ratio was assigned to ability to work under pressure and adapt to changes (98.55% and 95.19% of respondents described it as: an average important, important or very important, respectively) [26]. Stress is one of the environmental adaptations factors nowadays which have an increasing impact on the nervous system functions and homeostasis (e.g., endocrine functions of the adrenal glands and the pituitary-adrenal axis), which is associated with an increase in the concentration of catecholamines and glucocorticosteroids [100]. The presence of glucocorticoid receptors in the cellular components of the immune system is well described [101,102,103,104,105]. The relationship between the nervous and the immune systems is also well described in the literature, and the changes to the horizontal functioning of the nervous system that develop under the influence of prolonged and/ or pulsatile stimuli are one of the main factors correlated with the occurrence of diseases in civilizations, e.g., autoimmune etiology diseases. The Canadian physician Hans Seyle was the first to describe the anatomy of stress and stressors. He conducted extensive research and observations on the long-term stress and physical and mental changes it wrought. Specifically, the observed changes had adaptive qualities, and he named them general adaptation syndrome (GAS) [106]. Additionally, he researched the influence of a stressor on the precise point of its action, which he termed a local adaptation point. According to Seyle [106], GAS develops in three phases. The first is an alarm reaction stage during which the body mobilizes itself against stress factors. This phase results in the body entering a defensive mode that leads to an increase in blood pressure. The second phase is the shock resistance stage, which activates defense mechanisms through a change in physiological parameters during which the body enters into “fight mode”. The sympathetic nervous system activates, and adrenaline, noradrenaline, and cortisol are released. Blood pressure increases, breathing quickens, and the heart rate rises. Additionally, increased sweating occurs. The gastrointestinal system slows down. All those changes are general, independent of the kind of stressor. The next phase is the resistance stage during which the body resists the stressor until the perceived danger passes. The last stage is an exhaustion stage, in which the defensive mechanisms ceases fighting the stressor due to the harm associated with prolonged activation of the stress-related physiological functions. Hans Selye observed changes in energy during stress, calling it “adaptive energy”, and he divided this into superficial and deep. After activation, the body depletes the superficial energy first, and the energy gap is refueled by deep energy, which leads to adaptive changes in homeostasis, which can result in premature cell aging and death. In a stress reaction, the central nervous system, prefrontal cortex, corpus callosum, thalamus, and hypothalamic-pituitary-adrenal (HPA) axis all take part [106]. 

The HPA axis prepares an organism for an optimized and controlled stress response, and includes feedback regulation. Through thalamic activation, corticotropin-releasing factor (CRF) and vasopressin are released, influencing the release of corticotrophin, which is transported by the blood stream to the adrenal cortex, which then releases cortisol, cortisone and corticosterone [107,108]. 

Cortisol is a hormone involved in stress responses. It plays an important role in a decision-making process and shapes emotional memory [109]. Successful cortisol release is dependent on the connection between hormone and its receptors: glucocorticoid receptors (GR) and mineralocorticoid receptors (MR). GF receptors occur in any type of cell, while MR are located only in heart cells and certain populations of brain neurons [110]. At physiological cortisol concentration, MR are saturated, while cortisol bind to GR receptors only during stress situations [111,112]. Binding to GF receptors and its transport are associated with one of the designated FK506 binding protein-5 (FKBP5) variants. In homozygotes with single AA alleles, the influence of cortisol on its target cells is decreased, leading to negative feedback which results in prolonged release in stressful situations. This may contribute to many psychiatric and neurological disorders [109,113]. Increased stress levels can influence receptor expression. Cytoplasmic GR migrate to the nucleus after hormonal binding, working as transcription factors. An increase or decrease in gene expression can occur, depending on receptor and location. GR expression is decreased in various tissues [114], while, in immune cells, GR mRNA expression increases after administering dexamethasone [115]. GR can also regulate genes promotors. The *CRH* gene promotor in the HPA axis can be silenced in one tissue and stimulated in another [116]. Those dependencies stimulated by stress influence memory formation and learning process. Only some brain regions, like the hippocampus, are engaged in analyzing information associated with stress. In order to strengthen memory while in a stressful environment, neurons can be modulated differently by glucocorticoids than neurons located in other regions. Its effectiveness can be reflected not only in neuroendocrinology but also in behavior. Glucocorticoids affect many different cell types. Their differential effects on hundreds of target genes means multiple signal transduction pathways can be affected with associated different stress adaptation responses [117,118].

Norepinephrine and dopamine modulate communication between the limbic system and the prefrontal cortex. The degree of synaptic activity is mediated by catechol-O-methyltransferase (COMT) [119]. Research on *COMT* gene polymorphisms at the Val158/108Met position showed that Val/Val polymorphisms were associated with faster synaptic breakdown of dopamine. On the other hand, the Met/Met variant had increased sensitivity to the environment reflecting early-life adversity, but such a relationship not observed in Val/Val homozygotes [120]. Blunted cortisol reactivity in Met/Met carriers exposed to early stress could impact processes that depend on normal cortisol regulation following periods of acute stress [120,121].

The influence of long-term stress can result in mental disorders like depression, suicidal ideation, and addiction. Many genes have been examined clinically, in order to understand their working mechanisms and identify effective treatments. The key influential genes, identified to date, are the serotonin transporter gene *HTTLPR*, receptor genes *1A, 2A, 2C*, and genes coding BDNF, COMT, ACE, and TPH [122,123,124,125,126].

Skalkidou et al. [127] analyzed 94 polymorphic genotypes of the SNP type in 16 genes: *CRH*, *CRHBP*, *CRHR1*, *CRHR2*, *CYP17A1*, *CYP21A2*, *FKBP5*, *HSD11B1*, *HSD11B2*, *HSD3B1*, *HSD3B2*, *MC2R*, *NR3C2*, *NR3C1*, *POMC*, *SERPINA6*, whose protein byproducts are involved in the stress response of the HPA axis and influence stress in pregnant women and post-partum depression. Changes in SNP in *HSD11B1* and *SERPINA6* genes turned out to be statistically important in self-reported self-esteem results and depression prevalence. Women were examined in the 17th and 32nd weeks of their pregnancies and in the 6th week and 6th month after childbirth. During pregnancy, the greatest self-depression scores were found in women with the GG genotype in the rs1256540 position of *HSD11B1* gene. The same correlation was recorded postpartum. In *SERPINA6* rs8022616 gene, the GG genotype scored the highest and AA the lowest, also both pre- and postpartum [127]. 

Stress, as an environmental factor, shapes personality and leads to physiological changes, and excessive and chronic stress leads to many illnesses, both mental and somatic. Resistance to stressful work conditions is a biological adaptation, and genetic predispositions can make it easier or more difficult.

## 6. Human Emotions and Genotype

The negative connotations of emotions in the field of accounting and business are the basis for ignoring them in research and educational purposes. This is also due to the concept of accounting education focused on cognitive development [128,129] and analytical skills [130]. However, emotions are one of the key factors in decision-making processes. Similarly, accounting decisions can be based on poor or underdeveloped reasoning, so also decisions can be made based on poorly developed emotional intelligence [131,132,133]. According to McPhail’s study, accountants should be encouraged to take decisions based on their feelings (because they inevitably do anyway) [133]. He also argues that in order to do this critically, accountants need to have a more developed level of emotional intelligence [133]. According to the literature data, the emotional intelligence is one of the important predictor of success in accounting, like both technical skills and cognitive intelligence [131,133,134]. The genetic impaction of human emotions can be also one of the tools used to create a novel approach for building a teaching program for accounts and may be used to better understand the biological background related with job preferences and education of professional competence needed in financial sector.

Personality-building traits have individual stability, meaning that the same action will elicit the same reaction. Trait stability, or “level” of such emotions as anger, is not only regulated by environmental factors, but it also has a genetic background. Genes establish character and many behaviors, and environment changes and shapes traits in boundaries created by the genotype. Personality traits are 44% heritable, with environment influence constituting 55%. Twin studies have shown inheriting personality traits on different levels, evidencing that genetics play an important role. The correlation coefficient of personality traits for monozygotic twins is 0.50, while for dizygotic twins it is around 0.20 for children raised either separately or together [135,136].

Human behavior as a whole regulates emotions. In recent years, not only geneticists but also psychologists have begun to express interest in the oxytocin OXTR gene. Oxytocin is a neurohormone released directly into the bloodstream by neurons of the posterior pituitary gland [137,138,139]. The biological role of this hormone has been well researched and described during the last few decades, but new neuropsychological discoveries have shed new light on the relationship between hormone excretion and behavior. Oxytocin plays an important role in forming social habits such as attachments (as between a mother and child) [140], in expressing optimism and self-appreciation, and in reducing levels of aggression, fear, and stress [141], and in trust-building. A medication based on oxytocin is often administered to people who suffered child abuse to restore social bonds and the ability to trust [142,143]. During a test when subjects were administered doses of oxytocin, these subjects perceived faces displayed to them in photographs as being relatively friendlier and more attractive compared to subjects from the control group, who administered a placebo [144]. Saphire-Bernstein et al. [145] examined genetic polymorphism in OXTR gene and showed that subjects possessing two GG alleles had lower optimism, self-esteem, and belief in their own abilities to influence their behavior and environments. According to the research of Ohtsubo et al. [146], subjects with GA and GG alleles were more prone to self-inflicted punishment and reward deprivation than ones with AA alleles.

Vasopressin opposes many actions of oxytocin. Vasopressin is released from posterior pituitary nerve terminals and acts on vasopressin receptor 1A, encoded by the AVPR1a gene. This neurohormone heightens stress, fear, and aggression. Vasopressin influences neurons in the central nucleus of the amygdala responsible for fear responses. Elevated vasopressin levels can be triggered by increased osmotic pressure, increased sodium concentrations in the cerebrospinal fluid, hypovolemia, central nervous system (CNS) activation due to stress, and nicotine [141]. In 2008, AVPR1a gene was dubbed the “dictator gene”. Scientists from the Hebrew University of Israel working under Richard Ebstein discovered a connection between AVPR1a gene and the character traits callousness and egoism [147]. They used the economics game “Dictator”, in which a subject is forced to assume a selfless or a selfish role. Sadly, this research did not answer the question of whether gene polymorphism directly influences levels of cruelty or selfishness [148].

Genetic differences in the MAOA gene, which codes for monoamine oxidase-A, is a good example of a gene that influences fear responses. Wang et al. [149] showed that 40% of the fear of learning mathematics and solving math problems in children under 12 is genetically determined, and Liu and Lu [150] confirmed an association between polymorphisms in the MAOA gene and experiencing fear.

Not only do neurotransmitters induce certain emotions (like fear, depression, happiness, or joy), but abstract thinking enables the influence of the changes on human behavior to be understood and interpreted. Here, two genes are worth discussing–the dopamine receptor DRD genes: DRD2 and DRD4. Ben-Israel et al. [151] researched polymorphisms in 3rd exon of the DRD4 gene (DRD4-III) characterized by a repeat region of 48 base pairs (translated to 16 amino acids) that can be repeated 2–11 times. It has been shown that, among Caucasians, the second most common gene repetition is 7R or a sevenfold repetition of an allele that is associated with experiencing empathy and sensitivity. Adult women who carry the R7 allele have higher cognitive empathy than ones without it. It has also been shown that this correlation is reversed in adult males, meaning that this polymorphism is related to gender [152]. In children aged 3.5 and 5 years, respectively, a study focused on level of affective knowledge in two age and gender subgroups. Male children with DRD4-7R scored higher in affective knowledge than did female children in both age groups [83]. Absence of the 7R polymorphism in the gene eliminated the gender differences. Mayseless et al. [153] have found 7R polymorphism to be associated with decreased divergent thinking, especially flexible thinking. The DRD4 gene polymorphism also correlates with sensory sensitivity levels and is also gender related. In women, a short gene variation (up to 5R) decreases sensory sensitivity level, while a long gene variation (11R) is associated with an increased level of this sensitivity. In men, this correlation is reversed; polymorphism in exon III of the DRD4 gene is related to altruism levels. In addition, variability in gene receptors and serotonin and dopamine transporters has been found to affect level of vitality and life activity [83,154]. The other DRD2 dopamine receptor type is related to emotional intelligence. The appearance of CC genotype (g.67543C>T) in DRD2 gene correlates with decreased intellect and had an adverse influence on working memory in examined subjects [155].

Polymorphisms associated with Cloninger’s psychobiological model has shown a relationship between serotonin’s transporter promotor and a temperament that directly seeks to avoid harm [156]. Polymorphisms of the serotonin 5HT-2C receptor gene is associated with susceptibility to anxiety disorders and depression [157,158]. Neuroticism, a mental imbalance associated with fear perception, most probably has its roots in the coupling of certain genes with markers in the chromosomal areas 1q, 4q, 7p, 12q, and 13q. BDNF also appears to influence the phenotype associated with the neurotic personality disorder [159,160,161,162]. Interaction with the Val66Met polymorphism of the BDNF gene was reported in those phenotypes [159]. Biological function disorders or gene mutations can lead to severe personality disorders, i.e., “hypersensitivity to functioning”. It may result in an individual being overly concerned with details, having a high fear threshold, or experiencing a decision-making impairment [82,163].

Such disorders are hereditary and can be divided into three categories. The first one consists of unusual, paranoid and schizoid behaviors, emotional behaviors comprise the second one, and the third consists of anxiety behaviors. Antisocial personality originates in a genetic factor, not in external influences. Research has shown that the risk of antisocial behavior is five times greater in first-degree male relatives regardless of their place of upbringing, and ten times greater in women. 40% of male convicts and 8% of female convicts display this personality type. The compatibility of criminal traits in identical twins is near 52% and, in fraternal twins, is only 21%. Those suffering antisocial personality disorders display greater susceptibility to addictions [82,163].

## 7. Intelligence and Genotype

Intelligence is a fundamental trait not only in economics but in any discipline that demands the ability to think quickly, logically, and strategically. Integration of cognitive functions and abilities is dependent on the very general mental ability called general intelligence. It is a system of functional processes (memory, attention, perception, thinking, and problem-solving abilities) shaped by environmental factors so as to aid an individual in better adjusting to its habitat (therefore, it is also called emotional intelligence) [164]. Hans Eysenck stated that intelligence consists of the following basic cognitive functions: operating on numbers, speech fluency, observation, memory, and reasoning [165,166,167]. Howard Gardner [168] established a clear distinction between logical, emotional, mathematical, musical, linguistic, and spatial intelligences (i.e., multiple-intelligence theory). Can intelligence, as a source and beginning of every action, be written in the genome, or is it just an effect of time devoted to self-improvement? Are the genes the reason that one individuals learns quickly, whereas another learns slowly and with difficulty? 

Numerous data evidence the anatomic or neurophysiological implication and correlation related with general intelligences level (scores). For example voxel-by-voxel (a voxel is a volume element analogous to a pixel) analyses also showed specific areas where the amount of gray and white matter was correlated with intelligence scores [169,170,171]. Some research data suggest that cognitive processing, predicts value-based decision-making amongst older adults, while general intelligence, which relies on previously acquired knowledge, does not [169,171]. From the biological point of view, a key important predictor of intelligence have a genetic background modulated by the environment.

In the mid-19th century, an English scholar, F. Galton, a close relative of Charles Darwin, asked for the first time, “What shapes a human, genes or upbringing, nature versus nurture?” Galton did not expect his question to be the cause of intense disagreement among other scientists. He was the first to use a twin study and genealogical studies in his research, which caused him to create the theory displayed in his most famous work Hereditary Genius. In it, Galton states that intelligence is a heritable trait, and he sought to demonstrate this in several further studies. Galton also published two articles concerning the hereditary transmission of higher intelligence and other traits in which he concluded that all high-level traits are hereditary. Galton’s focus on heritability as the main factor shaping personality led to the creation of eugenics. Still another important event on this timeline is the formulation of Spearman’s theory, which discovered the “g factor” (general factor); a construct associated with processes such as deduction and reasoning, it was the first technique based on factor analysis. Similar conclusions were derived by French psychologist and intelligence quotient (IQ) scale creator Alfred Binet. He created the first test that sought to define “intellectual range” and was used to select intellectually-challenged pupils who were deemed not suitable to attend classes with average children. It resulted in the creation of a whole series of IQ tests suitable for various age groups. The following categories are based on these results: mental handicap (under 69), lower limit (70–79), under average (80–89), average (90–119), high (120–130), and very high (above 130) [135].

The 20th century saw dramatic advances in hereditability theories. The focus of these was mainly on surveying children and determining the extent of both environmental and genetic influences. This research was led by Plomin, who proved that upbringing (e.g., family values, time focused on learning) has a significant influence on development of intelligence but that genetics is equally important with respect to level of intelligence, which is hereditary. Children from the same parents tend to be very similar intellectually, even if they are brought up separately, in foster care for instance, and so most intellectual and mental abilities are heritable [172,173,174,175]. High intellect is also heritable and caused by the same factors as the ones that influence development of average intelligence [33]. Strelau modeled the roles of intelligence and environment in behavior with the formula B = f (GxE), where B signifies behavior, G genetic factors, and E environmental factors. If either of those factors had a value of 0, no behavior could occur [135].

Mono- and dizygotic twin studies uncovered significant differences in IQ correlations stemming from genetic sources and their minimal variability between early and middle childhood, while a greater variability was observed between adolescence and maturity. Heritability increases with age because genetic influences cause stronger phenotype effects as we age, prompting Plomin to state that “we grow into our genes” [175,176,177,178,179]. In his research, Bouchard [180] points out that intelligence heritability level reaches values of 50% or even 80%. Comparing genetic relationships with intelligence levels, one can notice a different correlation depending on degree of kinship. Monozygotic twins share 100% of their genes (but the correlation between intelligence and common genetic factors is *r* = 0.85); dizygotic twins share 50% of genes (*r* = 0.60); and first-degree relatives share 50% of genes but exhibit a correlation of only 0.45. Second-degree relatives have 12.5% of genes in common with one another (*r* = 0.15), whereas individuals that are unrelated share 0% of genes, and so have a correlation *r* = 0 [174].

One of the first tests [181] to measure cognitive development in twins and their siblings aged 3, 6, 9, 12, 18, 24, 30, and 36 months and 4.5 and 6 years showed the existence of a standard heritability pattern. Monozygotic twin correlations have risen systematically from 0.66 when aged three months to 0.85 at six years of age. Dizygotic twin correlations remained at 0.67 over these years, however estimated heritability increased from 0% after three months to 44% after six years. Monozygotic twins displayed less variability between them than did dizygotic twins of any age. Additionally, Wilson documented a similarity in development between twin pairs, comparing variability in mono- and dizygotic twins at ages 1 and 3 years [181]. In any period, the abovementioned variations in monozygotic twins was more highly correlated with age than in dizygotic twins, and variability between the pairs was significantly higher between dizygotic than monozygotic pairs. The correlation between mental development, birthweight, and gestational age in all twins decreased as chronological age increased, from 0.50 and 0.48 to 0.8 and 0.11 for 1-year olds and 3-year olds, respectively. The correlation between a child’s mental development and the mother’s education and father’s social status consistently rose from 0 to 0.35 [181].

People differ not only by intelligence but also by level of academic motivation, pleasure from learning, and understanding themselves through subjects they study in school. Kovas et al. [182] examined around 13,000 twins aged 9–16 from six countries, and their results showed that motivation, pleasure from learning, and perception of aptitude through study of varying subjects were also heritable. Moreover, the environment children were placed in did not influence their motivation with respect to learning. Genetic factors had a 40% influence [182]. Results from the research of Deary et al. [183] concerning exam passability in selected academic subjects showed a positive correlation (r = 0.81) with researched genetic traits in a population. Along with development, the brain undergoes morphological changes as an individual matures [184,185]. In the case of many adult brain structures, the volume of gray and white matter, the upper frontal and temporal lobe cortex, corpus callosum density, amygdala, and hippocampus, Broca’s area, the transverse temporal gyrus, to name the key brain regions influenced by genetic factors with 70–90% variability [186,187,188,189,190]. Similar data pertain to brain functions associated with intelligence, like dynamic brain oscillatory complexity [80]. The National Health Institute in America conducted a longitudinal study involving typically and atypically developing children, recruiting subjects between 5 and 18 years old and taking measurements every two years between these ages. The white and gray matter, caudate nucleus, and volume of the front and central part of the corpus callosum exhibited genetic influence consisting of 77–88% of the whole variation. Genetic influences on the volumes of the cerebellum and lateral ventricle were lower than 49% and 31%, respectively. Common environmental factors were few in number.

Intelligence, a complex, polygenic heritable trait, is determined not by one gene but by a whole gene group and varies according to environmental factors. Researching polymorphic changes in many genes, both in coding (exons) and non-coding (introns) parts, led to this conclusion. More specifically, the basis of this information lies in research on point mutations concerning a single nucleotide in a whole sequence, a so-called SNPs, which results from inertia or deletion and is the most common mutation in the human nuclear DNA that is responsible for variability. Despite intelligence being determined by the cooperation of many “small genes”, in some cases one mutation of a gene can have serious consequences, causing mental impairment (a low IQ in the range of 20–40), as in phenylketonuria and the PAH gene mutation, for instance. DNA marker research has described many candidate genes that display a lesser or greater correlation with intelligence levels. Some of them are presented in Table 1.

Plomin et al. [172] studied DNA markers that are directly associated with intelligence, surveying Caucasian children aged 6–12 with low, average, and high IQs. He examined 46 markers with two alleles and conducted 26 comparisons for 14 markers with two alleles. Five of 46 markers exhibited significant differences in frequency: *ESR*, *HLA-A (B)*, *INSR (B)*, *SOD2 (B)*, and *TA*. Moreover, three of 26 comparisons were significant with respect to DM, FMR-1, and MAP1B (A) markers. Based on this study and analysis, Plomin et al. [172] concluded that genes related to intelligence occur in gene pool of the central nervous system. Year later, in another research, Plomin et al. [173] examined another marker pool. The material was sourced from 276 twin pairs with low, average, and high IQs. To mark gene polymorphisms, Plomin et al. used PCR-RFLP, examining 36 markers with two alleles and making 11 comparisons for four markers with two alleles. Of 36 double-allele markers, three exhibited significant differences (*p* < 0.05) in frequency with respect to high and low IQ: *ADHS*, *EST00083*, and *NGFB*. Differences in frequency were also found in allele 4 of the apolipoprotein (APOE) gene, which had a higher frequency in the low-IQ group. In allele 3 of *APOE*, a higher frequency was observed in groups with higher IQs. APOE is a protein that is responsible for transporting lipids and involved in neuron reconstruction and that also prevents beta-amyloid buildup. Three variants of this gene (2, 3, 4) have been reported to date, with variant 4 associated with Alzheimer’s disease etiology. Research has shown that the presence of allele 4 in humans increases the risk of incurring this disease by three to four times and, in homozygote form, by as much as 10 times [194]. The cause of the disease is a beta-amyloid buildup in the brain due to APOE4′s not breaking it down properly. This buildup influences and disables brain functions, contributing to, among many other effects, long-term memory loss [195].

A beneficial correlation with intelligence levels has been observed in polymorphisms of *A31G* and the *G128T* gene promotors for the cholecystokinin A receptor (CCKAR) gene. This peptide hormone plays an important role in the gastrointestinal system and the central nervous system through neurotransmitter interactions. IQ levels associated with the presence of haplotype GA were the highest, and IQ levels associated with that of haplotype TG were the lowest [193].

The genes coding dopaminergic (*DRD2*, *DRD4*, *COMT*, *SLC6A3*, *DAT1*, *CCKAR*) and adrenergic (*ADRB2*, *CHRM2*) receptors, neutrophins (e.g., *BDNF*), and the genes encoding proteins that neutralize free radicals (*LTF*, *PRNP*) are considered an important group determining rational thinking. Genetic polymorphisms in c.957C>T of the *DRD2* gene, c.472G>A of the *COMT* gene, and c.46A>G of the *ADRB2* gene have shown to be beneficial with respect to increased intelligence [196].

Genes of neutrophins are also associated with the development of cognitive abilities such as perception, memory, and attention span. Neutrophins exert a large influence on the size of neuronal branching, ensuring correct neuronal morphology and the survival of cholinergic neurons. BDNF has also been shown to be associated with intelligence levels, involved in the development of serotonergic pathways, neocortical development, and hippocampus neurogenesis [197,198]. Additionally, it influences the learning process through long-term potentiation. The *BDNF* gene occupies the 11q13 position on short chromosome 11 arm [160]. It contains 11 exons and promoter sequences, whose activation depends on their placement [161]. Egan et al. [159] discovered that the g.27009G->A (c.472G->A, p.Val66Met) polymorphism in the *BDNF* gene influences episodic memory in schizophrenic patients. The position change of valine to methionine in the protein primary structure negatively influences hippocampal activity, resulting in functional impairment. Individuals with the above-mentioned point mutations tend to have worse memories than ones with unaltered proteins. Other research has shown that homozygote individuals (Val/Val) have higher intelligence levels than heterozygotes (Met/Val) [162]. A protein byproduct of the CHRM2 receptor gene is involved in the cognitive functions of attention and memory. The *CHRM2* gene is located on chromosome 7. Two independent studies on the single polymorphism c.1890A->T (g.152910A>T) in the non-coding part of the gene showed a positive correlation with intelligence level [199]. Thymine substitution at the aforementioned polymorphism g.152910A->T [200], 145263 (g.145263T->A, and c.46-5906T->A and guanine substitution in 140598 (g.140598G->A, c.46-10571G->A) and position 113002 (g.113002G->A, c.124-15534G->A) have been shown to correlate positively with nonverbal intelligence levels within subject groups. All described polymorphic changes are situated within non-coding regions of the M2 receptor gene a113002G->A in intron 4, and 145263T->A and 140598G->A in intron 5 [201].

A multiplex approach helps to quickly research a complex spectrum of polymorphic variabilities in many genes. DNA microarrays allow research of 700 000 polymorphic SNP-type places. The use of a genome-wide complex trait analysis (GCTA) method in research involving 3000 unrelated children resulted in establishing a connection between family socioeconomic status and children’s IQ [179].

Additionally, during screening tests, Harris et al. [202] established a dependency between intelligence level and functionality and variability in protein-coding genes engaged in oxidative stress defense mechanisms, research that resulted in the identification of over 300 SNPs in over 100 genes. Davies et al. [203] conducted whole-genome analysis involving 3511 individuals, analyzing 549692 SNPs together with detailed phenotype cognitive traits. Despite no genetic variants being found to be strongly correlated with human intelligence levels, these results showed that the major part (~40–50%) of variability in human intelligence is associated with common SNP and led to the conclusion that longer chromosomes explain greater variability in intelligence.

Research conducted on SNP and on selected genes has confirmed the influence of genetics on children’s intellectual development and has lent credibility to the hypothesis that there are sets of genes with small individual effects but significant synergy in shaping intelligence. Researching intelligence-associated genes has often been sparked by an interest in mental disorders and disabilities [204], aging [183,203], and mental disorders like schizophrenia [205]. Some genes which may be significant with respect to intelligence are associated with brain size (e.g., *ASPM* and *MCPH* genes), dementia (e.g., *APEO*) [206], dopamine system (e.g., *COMT*) [121] longevity (e.g., *KLOTHO*) [183] or oxidative stress (e.g., *PRNP*) [207].

Benjamin et al. [208] used the genomic relatedness matrix restricted maximum likelihood (GREML) technique to research preferred phenotype traits in economic vocations. Four basic economic preferences were included: reluctance to assume risk, trustworthiness, patience, integrity, and academic success. The GREML technique uses dense SNP data to estimate the proportion of variance in traits that, taken together, can be explained by the analyzed SNPs. According to their research, trust was the only one of the four examined features found to be statistically significant (*p* = 0.047), suggesting that the common SNPs explain over 20% of phenotypic variation. Additionally, research was conducted in Australia to determine the effects of genetic and environmental influences on academic achievements, employing a multiple regression model [209]. “This common model is using [sic] to estimate environmental and genetic components of variance in both population-based samples of twins and samples where proband members of twin pairs were selected for having extreme scores exceeding a design threshold” [210]. The results of this research point to the huge influence of genetics on academic achievement on a 50% variation level, with common familial factors having a 25–50% level, depending on the research method employed [211].

Research on finding the next gene we can call “an intelligence gene” is being conducted continually. In 2012, the *HMGA2* gene coding the non-histone chromatin protein was named one of these. Scientists from the University of California led by Thomson as part of the ENGMA (Enhancing Neuro Imaging Genetics through Meta-Analysis) project tested 21,000 individuals, analyzing MRI images of the brain and *HMGA2* gene polymorphism. The results determined that, in mutated alleles and in ones where thymine was substituted by cytosine, IQ increased by 1.29 points and brain volume increased in 0.58%. Discovering the *TESC* gene version, which influences the volume of the hippocampus, was also a key achievement of Thompson’s team [190,212].

Genetics determines IQ variability, especially in the upper part of the scale, and environmental factors can modify this range positively or negatively. Although we are born with predispositions or skills, it is within our power to decide in which direction these skills will be developed. On one hand, a person having a better genetic basis for a high IQ may never reach this level if his or her environment is not supportive, and, on the other hand, an individual without “good genes” can reach high intelligence levels due to hard work. A similar situation can occur with respect to mental disability. In some cases, a biological barrier is, on some level, unavoidable.

## 8. Conclusions

It is worth noting the wide-ranging population-based research needed to look for genetic markers of desired features in different types of employees in the financial sector. This research could help to better understand the biological mechanisms underlying the formation of certain phenotypes. Individual variability depends on environmental factors to more or less the same extent as personal genotype. Therefore, the genotype manifested in the phenotype is formed by the perpetuation of molecular changes and is translated into a unique set of biological features. Epigenetic changes are manifested by intracellular, variable nuclear DNA methylation patterns, as well as post-translational modifications of histone proteins (e.g., acetylation), which translates into the level of expression of cellular proteins or even complete lack of expression of selected proteins. From this point of view, genetics gives only a background to create a personality. Thus, it is nearly impossible to find a particular genotype needed to be a better accountant, banker, or politician; however, the knowledge of an individual’s genetic background can help to create more effective training tools and contentedness in the workplace.

It is important to use the knowledge from wide range of scientific disciplines (including economics, psychology, behavioral genetics) to create background to pursuit multidisciplinary research models in the field of neuroaccounting. This would help in pointing the best genetically based behavioral profile of future successful financial and accounting specialists. Thus, the multidisciplinary approach to creating research models will facilitate the creation of new guidelines in the development of occupational hygiene assumptions, the creation of more effective tools for professional training and the development of professional selection tools.

## Figures and Tables

**Table 1:** List of genes associated with human intelligence [172,173,191,192,193].

Gene	Protein	Chromosome Localization
*ADH2*	alcohol dehydrogenase 2	4q21–q23
*ADH3*	alcohol dehydrogenase 3	4q21–q23
*ADH5*	alcohol dehydrogenase 5	4q21–q25
*ADRB2*	beta-2 adrenergic receptor	5q33
*AGXT*	alanine-glyoxylate and serine-pyruvate aminotransferase	2p36–q37
*ALPP*	alkaline phosphatase, placental	2q37
*ALPL*	alkaline phosphatase	1p36.1–p34
*APOE*	apolipoprotein E	19q13
*ATP1B*	sodium-potassium ATPase subunit beta 1	1q22
*ALDH5A1*	aldehyde dehydrogenase 5 family member A1	6p22
*ASPM*	abnormal spindle-like microcephaly-associated protein	1q31
*BDNF*	brain-derived neurotrophic factor	11p14
*BPI*	bactericidal permeability-increasing	20q12
*CTG-B37*	atrophin-1	12p
*CHRM2*	cholinergic receptor muscarinic 2	7q33
*CD27*	CD27 antigen	12p13
*CCKAR*	cholecystokinin A receptor	4p15
*CTSD*	cathepsin D	11p15
*CHRNA7*	cholinergic receptor nicotinic alpha 7 subunit	15q14
*CYP2D6*	cytochrome P450 family 2 subfamily D member 6	22q13
*COMT*	catechol-O-methyltransferase	22q11
*CKB*	brain-type creatine kinase also	14q32.3
*DTNBP1*	dystrobrevin binding protein 1	6p22
*DISC1*	disrupted-in-dchizophrenia 1 (DISC1) scaffold protein	1q42.1
*DAT1*	dopamine transporter 1	5p15.3
*DM*	myotonic dystrophy	19q13.3
*MCPH1*	microcephalin 1	8p23
*DRD1*	dopamine receptor D1	5q34–q35
*DRD2*	dopamine receptor D2	11q23
*DRD3*	dopamine receptor D3	3q13.3
*DRD4*	dopamine receptor D4	11p15
*EST00083*	cDNA sequence from hippocampal library	15926 bp mtDNA
*ESR*	estrogen receptor	6q24–q27
*FADS2*	fatty acid desaturase 2	11q12
*FBN1*	fibrillin-1	15q15
*GLUT2*	glucose transporter 2	3q26 1–q26.3
*GLUT3*	glucose transporter 3	12p13.3
*GLUT4*	glucose transporter 4	17p13
*GAD1*	glutamate decarboxylase 1	2q31.1
*GAA*	acid alpha-glucosidase	17q23
*GRL*	glucocorticoid receptor	5q31–q32
*Hsp70*	heat shock protein	6p21.3
*HEXA*	beta-hexosaminidase A	15q23–q24
*HEXB*	beta-hexosaminidase B	5q13
*HOX2F*	Homeobox 2F	17q21–q22
*HOX2G*	Homeobox 2G	17q21–q22
*HTR2*	serotonin-receptor 2	13q14–q21
*HTR2A*	serotonin-receptor 2A	13q14
*HLA-H*	major histocompatibility complex, class I, H	6p21.3
*HLA-A*	major histocompatibility complex, class I, A	6p21.3
*HP*	haptoglobin	16q22.1
*INSR*	insulin receptor	19p13.3–p13.2
*IGF2R*	insulin-like growth factor 2 receptor	6q26
*KL*	klotho	13q13
*LAMB1*	laminin subunit beta 1	7q31
*LAMB2*	laminin subunit beta 2	3p21
*MYH2*	myosin heavy chain 2	17p13.1
*MAP2*	microtubule associated protein 2	2q34–q35
*MAOA*	monoamine oxidase A	Xp11.3
*NRN1*	neutrin 1	6p25.1
*NGFB*	nerve growth factor-beta	1p13
*NEFM*	neurofilament medium polypeptide	8p21.2
*OXTR*	oxytocin receptor	3p25
*OTC*	ornithine carbamoyltransferase	Xp11.4
*PPP1R1B*	protein phosphatase 1 regulatory subunit 1B	17q12
*PCCA*	propionyl-CoA carboxylase subunit A	13q31–q34
*PCCB*	propionyl-CoA carboxylase subunit B	3q21-–q22
*PAH*	phenylalanine hydroxylase	12q22–q24.2
*PRNP*	prion protein	20p13
*PTH*	parathyroid hormone	11p15.2–p15.1
*PCI*	protein C inhibitor	14q32.1
*SELE*	selectin E	1q22–q25
*SOD2*	superoxide dismutase 2 [Mn], mitochondrial	6q21
*SNAP25*	synaptosomal-associated protein 25kDa	20p12
*S100B*	S100 calcium-binding protein B	21q22
*SLC6A3*	sodium-dependent dopamine transporter	5p15
*TIMP2*	tissue inhibitor of metalloproteinases 2	17q22–q25
*TAT*	tyrosine aminotransferase	16q22.1
*TG*	thyroglobulin	8q24
*TGFA*	transforming growth factor alpha	2p13
*TH*	tyrosine hydroxylase	11p15.5
*THRB*	thyroid hormone receptor beta	3p24.1–p22
*TPO*	thyroid peroxidase	2p25–p24
*UNG*	uracil DNA glycosylase	12q24
*WRN*	Werner syndrome ATP-dependent helicase	8p12
*VDR*	vitamin D receptor	12q12–q14
*ZNF40*	Human Immunodeficiency Virus Type I Enhancer Binding Protein 1	6p24.1
